# Does the Evidence Support the Existence of the Simian Polyomavirus SV40 Vp4 Viroporin?

**DOI:** 10.1128/mSphere.00019-20

**Published:** 2020-03-18

**Authors:** Stian Henriksen, Christine Hanssen Rinaldo

**Affiliations:** a University Hospital of North Norway, Department of Microbiology and Infection Control, Tromsø, Norway; b UiT The Arctic University of Norway, Department of Clinical Medicine, Metabolic and Renal Research Group, Tromsø, Norway; University of Michigan-Ann Arbor

**Keywords:** BK virus, BKPyV, SV40, Vp4, polyomavirus, simian virus 40, viroporin

## Abstract

The simian polyomavirus SV40 was reported to express Vp4, an N-terminally truncated form of the minor capsid proteins Vp2 and Vp3. Since a missense mutation of the putative Vp4 start codon (Vp2M228I) was found to give reduced progeny release and delayed lysis, Vp4 was claimed to be a viroporin. However, two independent research groups, including our own, were unable to replicate these findings. In contrast, we found no Vp4 expression in SV40-infected cells and no reduction in progeny release for Vp4-deficient virus, and finally, we found that the single amino acid substitution unavoidably introduced into the overlapping Vp2/Vp3 genes during Vp4 mutagenesis reduced early steps but not virus release. Remarkably, the existence of the viroporin Vp4 still seems to be widely accepted, which presumably is preventing important research on polyomavirus release. With this perspective, we will review and comment on the most important experiments that led to the disputed announcement of the viroporin Vp4.

## Abstract (precis)

The simian polyomavirus SV40 was reported to express Vp4, an N-terminally truncated form of the minor capsid proteins Vp2 and Vp3. Since a missense mutation of the putative Vp4 start codon (Vp2M228I) was found to give reduced progeny release and delayed lysis, Vp4 was claimed to be a viroporin. However, two independent research groups, including our own, were unable to replicate these findings. In contrast, we found no Vp4 expression in SV40-infected cells and no reduction in progeny release for Vp4-deficient virus, and finally, we found that the single amino acid substitution unavoidably introduced into the overlapping Vp2/Vp3 genes during Vp4 mutagenesis reduced early steps but not virus release.

## PERSPECTIVE

Infection with human polyomavirus is a growing challenge in immunocompromised patients. Whereas BK polyomavirus (BKPyV) replication in bladder and in kidney graft epithelial cells may cause hemorrhagic cystitis or nephropathy, respectively, JC polyomavirus replication in oligodendrocytes is a hallmark of progressive multifocal leukoencephalopathy (1, 2). Although the pathogenesis of these diseases is slowly unraveling, it is still unclear how the aforementioned nonenveloped DNA viruses exit their host cells. In 2007, the closely related simian polyomavirus SV40 was described to express a very late protein triggering lytic release (3). This 13.9-kDa protein, designated Vp4, appeared to be an N-terminally truncated form of the minor capsid proteins Vp2 and Vp3, corresponding to the 125-amino-acid C terminus. In contrast to Vp2 and Vp3, Vp4 was not part of the virion. Later, Vp4 was described to be a viroporin, forming aqueous pores that increased the membrane permeability (4–6). Based upon citations in papers and virology textbooks, the existence of SV40 Vp4 seems to be widely accepted, despite the conflicting data from independent research groups (7, 8).

With this perspective, we review the key experiments that led Daniels and colleagues (3) to conclude that Vp4 is a late protein that triggers the lytic release of SV40 and comment on why these data, in our view, do not constitute sufficient evidence of Vp4 expression and viroporin activity.

## THE *IN VITRO* TRANSLATION PROOF

When Daniels and colleagues performed rabbit reticulocyte translation of T7 transcribed SV40 Vp2 mRNA, subsequent SDS-PAGE revealed, in addition to the expected Vp2 (38.5-kDa) and Vp3 (26.9-kDa) proteins, a protein with a molecular weight of ∼15 kDa (3). Using a vertebrate translation initiation prediction server (NetStart 1.0) (9), they found that Vp2 mRNA contained three additional in-frame start codons. Surprisingly, the highest score was found for the putative start codon correlating with Vp2 amino acid position 228 (Vp2M228), which, if utilized, would give the protein Vp4 (∼13.9 kDa). The second-highest score was found for the Vp2 start codon, followed by a putative start codon at amino acid position 295 and finally by the Vp3 start codon. When they changed Vp2M228 from ATG (methionine) to ATA (isoleucine), the *in vitro* translation of Vp4 was prevented.

*In vitro* synthesis of proteins in cell extracts is normally used to synthesize proteins for characterization and not to prove their existence. Although viruses rely exclusively on the translation machinery of the host cell, it is important to bear in mind that viruses have multiple mechanisms to regulate their transcription and translation, not reflected by *in vitro* translation (10). For instance, viral late genes seem to be optimized for expression in their natural host cells using codons matching the available aminoacyl-tRNA repertoire (11). Moreover, viral expression may be dependent on virus-encoded transinducers (12). We have indeed experienced these restrictions. In order to recombinantly express BKPyV Vp2, Vp3, and Vp4 and SV40 Vp4 in different human cells, we had to either codon optimize the genes by using codons frequently found in human genes or add N-terminal tags like enhanced green fluorescent protein (EGFP) (reference 8 and unpublished results). This was in spite of the use of a plasmid with the strong cytomegalovirus immediate early promoter.

If the NetStart definition of probable translation start sites was strictly used (score of >0.5), SV40 19S mRNA would contain only a Vp2 and a Vp4 start codon. However, in SV40-infected cells, Vp2 and Vp3 are both made from the 19S mRNA, where Vp3 is synthesized from its own start codon independently from Vp2 (13, 14). Contrary to the translation initiation prediction by Daniels and colleagues, the Vp2 start codon has a weaker Kozak sequence than the Vp3 start codon (15), leading to leaky ribosome scanning (13, 14). Together with two internal ribosome entry sites upstream of the Vp3 coding sequence, this probably explains the higher expression of Vp3 than of Vp2 seen in SV40-infected cells (16). Apparently, NetStart is less accurate than other predicting methods (17). In our opinion, neither the *in vitro* translation nor the translation start codon prediction is sufficient evidence for Vp4 expression in SV40-infected cells.

## THE PROOF OF Vp4 EXPRESSION AFTER SV40 GENOME TRANSFECTION

Next, Daniels and colleagues generated a series of plasmids containing mutant SV40 genomes and transfected BS-C-1 cells (3). Five days posttransfection, nuclear extracts were harvested and Western blot assay using polyclonal rabbit SV40 Vp2/Vp3 antisera was performed. The wild-type genome and the Vp4-deficient mutant Vp2M228I both gave rise to two bands corresponding to Vp2 and Vp3. In addition, the wild-type genome gave a band corresponding in molecular weight to the putative Vp4 protein, while this band was dramatically reduced following transfection with the mutant Vp2M228I. Finally, following transfection with a mutant containing missense mutations in both the Vp2 and Vp3 start codons, the band corresponding to Vp4 appeared again. The latter result was taken as evidence of *de novo* Vp4 expression.

There are several examples showing that disturbance of upstream start codons might influence expression. An SV40 mutant producing 19S but not 16S mRNA could produce Vp1 if a large deletion was introduced upstream of the Vp1 start codon or if the Vp3 start codon was mutated (13). Moreover, *in vitro* translation of SV40 Vp2 was most efficient without an upstream start codon (13).

Importantly, when the wild-type genome is used for transfection, all bands observed below the Vp3 band may theoretically be degradation products of Vp2/Vp3. In fact, we have previously observed that the C-terminal part of Vp3 is prone to proteolysis (8). Despite the use of a sensitive liquid chromatography-tandem mass spectrometry (LC-MS/MS) method on Vp2/Vp3 pulldown from cell extracts and on N-terminally dimethyl-labeled nuclear extract, both from SV40-infected cells, we found no decisive evidence of SV40 Vp4 (8). Rather, our results suggested that the observed band was actually a degradation product. This would explain the reported late emergence, about 24 h after the late proteins (3).

Although the universal AUG codon is a common start codon for translation in eukaryotes, not all AUG codons are start codons. Similarly to SV40, the Merkel cell polyomavirus (MCPyV) has a potential Vp3 start codon inside and in-frame with the Vp2 gene. Even though this open reading frame can be recombinantly expressed, Vp3 has not been detected in the virion or in MCPyV-infected cells (18). Although we did not observe a probable Vp4 band after transfection of a BKPyV mutant with missense mutations of both upstream start codons (8), we do acknowledge that SV40 Vp4 may be expressed in such an artificial setting.

## THE PROOF OF Vp4 ROLE IN SV40 PROGENY RELEASE

Next, Daniels and colleagues investigated how different late-gene start codon mutations affected SV40 replication. In short, BS-C-1 cells were transfected with the different SV40 genomes including wild-type virus and the Vp4-deficient mutant Vp2M228I. At 2 days posttransfection, about 3% of all cells expressed large tumor antigen (LTag), demonstrating a low but identical transfection efficiency. However, at 7 days posttransfection, 65% of wild-type-transfected cells and only 10% of Vp4-deficient mutant Vp2M228I-transfected cells expressed LTag. At 12 days posttransfection, the complete cell population was lysed by wild-type virus, while the mutant virus took 24 days. When supernatants from the lysed cells were used as inoculum, LTag staining at 2 days postinfection revealed that the wild-type virus infected 95% more cells than the mutant virus. The interpretation of the results was that the mutant had a prolonged replication cycle due to a defect in viral release. One independent research group performing similar experiments supported this notion (19). Next, Daniels compared cell death in cells infected by apparently equal numbers of infectious virus based on LTag expression at 2 days postinfection. At 3 days after infection with wild-type virus, trypan blue staining demonstrated a doubling of dead cells (from ∼4% to ∼8%), while this was not found in mutant-infected cells. At 5 days postinfection, when Vp4 is supposedly expressed, ∼10% and ∼7% dead cells were observed, respectively. From these results, the authors concluded that SV40 Vp4 initiated efficient lytic progeny release.

According to Daniels and colleagues, the SV40 replication cycle initiated by transfection or infection of BS-C-1 cells was 2.5 to 4 days and 3 to 5 days, respectively (3, 20, 21). This means that at the time selected for LTag immunofluorescence staining, 7 days posttransfection, at least one replication cycle was completed and a second cycle was started. This implies that the number of infected cells observed was the result not only of the progeny release from transfected cells but also of the success of the virus in infecting new cells. Although the trypan blue staining experiments might seem to support an impaired release of the mutant virus, it is important to bear in mind that this crude method gives only an indirect measurement of cells that are dead or have a transiently disturbed membrane integrity (22). Moreover, numbers were low and statistics were missing.

We investigated progeny release for SV40 (strain 776) and BKPyV (Dunlop and WW) using three different monkey kidney cell lines, including BS-C-1 cells, and primary human renal epithelial cells, respectively, and highly sensitive direct methods (8). Quantitative PCR of DNase I-treated supernatants and Western blotting of virions pelleted from supernatants harvested at 2 days posttransfection for SV40 and at 3 days posttransfection for BKPyV revealed that the same amounts of wild-type and Vp4-deficient mutants were released, clearly demonstrating that Vp4 played no role in progeny release. In addition, we investigated viral entry/uncoating by inoculating naive cells with supernatants from transfected cells. The Vp4-deficient mutants SV40 Vp2M228I and the corresponding BKPyV Vp2M229I demonstrated a 43% and a 90% reduction in infectivity compared to wild-type virus, respectively. Of note, our results are in full agreement with the results of Tange et al. (7). They concluded that d-type mutant tsD222, which is identical to SV40 Vp2M228I, had a defect in the early steps.

In order to reduce the possibility of revertants and to explore the tolerance of the missense mutations unavoidably introduced into the overlapping Vp2 and Vp3 genes, we created a second set of Vp4-deficient mutants, by changing the Vp4 start codon to GCC (alanine) (8). SV40 Vp2M228A and BKPyV Vp2M229A were released from transfected cells at a similar level as the other viruses used. However, in contrast to SV40 Vp2M228I, SV40 Vp2M228A did not show reduced infectivity, while a 65% reduction was seen for BKPyV Vp2M229A. These crucial experiments revealed that the reduced viral yield observed by Daniels et al. (3), Tange et al. (7), Luo et al. (19), and us (8) was caused not by a lack of Vp4 but rather by a functional deficit in Vp2 and Vp3 caused by the replacement of one methionine by isoleucine. This isoleucine residue seems to have a strong negative effect on early steps of the viral replication cycle such as viral entry or uncoating. Replacing the methionine with alanine did not compromise the function of SV40 but had some negative effect on BKPyV. In addition, we investigated cell death by real-time experiments measuring electrical impedance of SV40-transfected cells and DNA staining in SV40-infected cells (8). The results clearly showed that cell death was completely independent of Vp4 expression. In our opinion, we have proven that a potential Vp4 protein has a negligible, if any, role in SV40 or in BKPyV progeny release under the conditions investigated.

## CONCLUSIONS

SV40 Vp4 may be expressed in the artificial setting of translation in rabbit reticulocyte extracts or after transfection with an SV40 genome without upstream start codons. However, we find no valid evidence for Vp4 expression in SV40-infected cells. By including a mutation control (Vp2M228A), we have demonstrated that a putative Vp4 protein has no role in progeny release or virus-induced cell death in this setting. Importantly, when specific steps in a viral replication cycle are investigated, it is crucial that the correct time points and methods are used. Failing to do so may lead to misinterpretations, as we believe was the case for Vp4-deficient virus, where a defect in early steps was interpreted as a defect in virus release. Is SV40-induced cell lysis after all only a consequence of viral protein overexpression, or is there another virus-regulated mechanism? The search is on.
